# Pre-Clustering of the B Cell Antigen Receptor Demonstrated by Mathematically Extended Electron Microscopy

**DOI:** 10.3389/fimmu.2013.00427

**Published:** 2013-12-06

**Authors:** Gina J. Fiala, Daniel Kaschek, Britta Blumenthal, Michael Reth, Jens Timmer, Wolfgang W. A. Schamel

**Affiliations:** ^1^Faculty of Biology, Department of Molecular Immunology, Albert Ludwigs University Freiburg, Freiburg, Germany; ^2^Spemann Graduate School of Biology and Medicine (SGBM), Albert Ludwigs University Freiburg, Freiburg, Germany; ^3^Centre for Biological Signalling Studies BIOSS, Albert Ludwigs University Freiburg, Freiburg, Germany; ^4^Institute of Physics, Albert Ludwigs University Freiburg, Freiburg, Germany; ^5^Medical Faculty, Centre for Chronic Immunodeficiency CCI, University Clinics Freiburg, Albert Ludwigs University Freiburg, Freiburg, Germany; ^6^Max Planck-Institute of Immunobiology and Epigenetics, Freiburg, Germany

**Keywords:** BCR, oligomerization, electron microscopy, immuno-gold-labeling, Monte Carlo simulation, maximum-likelihood method

## Abstract

The B cell antigen receptor (BCR) plays a crucial role in adaptive immunity, since antigen-induced signaling by the BCR leads to the activation of the B cell and production of antibodies during an immune response. However, the spatial nano-scale organization of the BCR on the cell surface prior to antigen encounter is still controversial. Here, we fixed murine B cells, stained the BCRs on the cell surface with immuno-gold and visualized the distribution of the gold particles by transmission electron microscopy. Approximately 30% of the gold particles were clustered. However the low staining efficiency of 15% precluded a quantitative conclusion concerning the oligomerization state of the BCRs. To overcome this limitation, we used Monte-Carlo simulations to include or to exclude possible distributions of the BCRs. Our combined experimental-modeling approach assuming the lowest number of different BCR sizes to explain the observed gold distribution suggests that 40% of the surface IgD-BCR was present in dimers and 60% formed large laminar clusters of about 18 receptors. In contrast, a transmembrane mutant of the mIgD molecule only formed IgD-BCR dimers. Our approach complements high resolution fluorescence imaging and clearly demonstrates the existence of pre-formed BCR clusters on resting B cells, questioning the classical cross-linking model of BCR activation.

## 1 Introduction

Cells communicate with each other and with their surroundings through transmembrane receptors that are embedded in the plasma membrane. Thus, it is of high interest to understand how these receptors and other cell surface proteins, such as adhesion molecules or channels, are organized on the membrane. Initially it was thought that proteins and lipids freely diffuse in membranes and that they are randomly distributed (1). With the concept of lipid rafts it was noted that specialized microdomains on the cell surface exist, where some proteins are concentrated and others are excluded (2). Although the raft concept had to be modified, since in biological membranes they are smaller and more transient than in artificial model membranes (3), it is clear that proteins are not randomly distributed on the cell surface. One example of the immune system is the T cell antigen receptor that can form pre-clustered oligomers, called nanoclusters, on T cells (4–7). Nanoclusters form before and independently of any ligand encounter. Interestingly, T cells can control the degree of TCR nanoclustering, in order to regulate their avidity toward multivalent ligands and thus their sensitivity (8–10). This indicates that studying the nano-scale distribution of a receptor contributes to the understanding of the function of the receptor. Less well understood is a potential pre-clustering of the B cell antigen receptor (BCR). The BCR is expressed on B cells and controls the development of these cells and their activation upon contact with the BCR’s ligand, called antigen. The BCR is composed of the membrane-bound immunoglobulin (mIg) molecule and a heterodimer of the Igα (mb-1) and Igβ (B29) proteins (11). The mIg molecule binds to the antigen and exists in different isotypes, of which the mIgD form is the most abundant one on resting mature B cells (12, 13). The Igα/Igβ dimer contains phosphorylatable tyrosines in the cytoplasmic tails (14) and transmits the signal of antigen-binding to the cytoplasmic signaling machinery. The first evidence for BCR pre-clustering, i.e., the existence of BCR oligomers, was obtained by Blue Native gel electrophoresis (15–17). Upon extraction of the IgD- and IgM-BCRs from the cell membrane of resting B cells using low concentrations of detergent, the BCRs were found in oligomers. Importantly, a mutant mIgD molecule, in which the transmembrane region was mutated (called mIgD-hSbap), only formed dimers (16). Thus, in the BCR as well as in the TCR (10), the transmembrane region of the ligand-binding subunits is involved in the pre-clustering. Later, three approaches were used to investigate whether the BCR forms oligomers in living cells. Firstly, a FRET approach was used, and oligomers were not detected (18). Secondly, a bifluorescence complementation approach was used, and BCR oligomers were detected (19). The differences in these two procedures and possible functional implications of pre-clustered BCR oligomers were recently discussed (20). Thirdly, the superresolution microscopy method direct stochastic optical reconstruction microscopy (dSTORM) was used to show that BCRs were organized as pre-clusters on the surface of resting primary B cells (21). Here, we used a validated technique, that we had previously used to study TCR pre-clustering (6, 8), in order to answer the question of whether BCR oligomers exist and if yes what their sizes are. To this end, we used fixed B cells and labeled the BCRs with specific antibodies that were bound to gold particles (immuno-gold-staining), prepared cell surface replicas and analyzed the nano-scale distribution of the gold particles by transmission electron microscopy (TEM). This approach allowed visualization of BCRs on cell surface areas that do not adhere to any experimental support, giving the opportunity to analyze untouched (and non-modified) receptors. A general challenge of immuno-gold-labeling is its low staining efficiency, which is compensated by mathematical modeling and statistical methods, allowing solid conclusions to be drawn from the experimental data.

## 2 Materials and Methods

### 2.1 Experimental procedures

#### 2.1.1 Cell culture and cell fixation

The murine B cell lines J558L (not expressing any BCR) and J558Lδm/mb-1flN (expressing an IgD-BCR) were previously described (16). We also used a J558L line that expressed a mutant IgD-BCR, in which the transmembrane region of the mIgD molecule was mutated (mIgD-hSbap) (16). Cells were cultured in RPMI 1640 complete medium supplemented with 10% fetal calf serum, 2 mM l-glutamine, 100 U/ml penicillin/streptomycin, 10 mM HEPES, and 50 mM 2-mercaptoethanol and grown at 37°C in a humidified atmosphere with 5% CO2. Cells were fixed with freshly prepared, ice-cold 4% paraformaldehyde in PBS for 20 min at 4°C at a cell density of 10 × 10^6^ cells/ml. After fixing, cells were washed twice with cold PBS.

#### 2.1.2 Immuno-gold-staining and analysis of gold-reagent

Unstimulated PFA-fixed cells were stained with the primary anti-idiotypic antibody Ac146 (22) at saturating concentration of 20 mg/ml in PBS with 1% BSA for 1 h on ice. This antibody binds to the variable regions of the BCR used in this study. Staining with a secondary anti-mouse IgG antibody conjugated to 10 nm gold (Aurion) was performed for 1 h on ice. Prior to cell staining, the aggregation state of the gold-reagent was tested by adsorbing diluted suspensions of the gold-reagent onto collodion/carbon-coated EM grids, which were analyzed in transmission electron microscopy (TEM, Figure 2).

#### 2.1.3 Surface replica preparation

Labeled cells were adsorbed to l-poly-lysine-treated micas, followed by a second fixation with 0.1% glutaraldehyde on ice for 30 min. Micas containing stained cells were covered with an untreated piece of mica and fast-frozen in a Reichert-Jung (now Leica) KF-80 plunge freezing unit using the secondary cryogen liquid ethane. Metal replicas were prepared in a freeze fracture unit (BAF 060; BAL-TEC) where the cell-containing mica slide was freeze-etched at −150°C for 12 min to sublime surface ice. Frozen cells were then shadowed with 2 nm of evaporated platinum at an angle of 45°C and strengthened by a uniformly thick 20 nm electron-translucent carbon layer evaporated perpendicular to the mica surface plane. The metal replica of the surface was released from the mica by floating it on commercial bleach where it remained over night for digestion of the organic material. The floating replica was washed three times in distilled water to remove attached organic material and chemicals and then picked up on uncoated copper EM grids. For a detailed protocol see Ref. (23).

#### 2.1.4 Analysis of metal replicas by TEM

Replicas mounted on EM grids were examined in a transmission electron microscope (1200-EX II; JOEL) operating at 100 kV. Gold particle numbers and the gold cluster size distribution were counted and analyzed for at least 3 cells per sample at an augmentation of 25000. Gold particles were considered to be part of the same cluster when they were adjacent or less distant than 10 nm (the diameter of a single gold particle), taking into account that the diameter of the BCR is around 10 nm (24). Pictures were taken at augmentations of 5000 (cell overview), 120000, and 300000 (gold-labeling).

#### 2.1.5 Quantification of the number of BCRs per cell

The number of BCRs per cell was determined based on a saturation binding assay. About 1 × 10^6^ J558Lδm/mb-1flN cells were stained for 30 min at 4°C with increasing concentrations of an FITC-coupled anti-IgD antibody (BD Pharmingen, clone 11-26c.2a). Following 5 extensive washing steps, the fluorescence signal was measured in duplicates using a SpectraMax 190 Absorbance Microplate Reader. In order to convert the fluorescence signal intensity into the number of antibodies, a calibration of the antibody was performed by fitting the model *y* = *mx* + *b* to the standard concentrations *x* and fluorescence signals *y*. Here *m* denotes the slope and *b* denotes the intercept of the calibration curve. In order to infer the number of receptors on the cells, antibody was spotted in different concentrations. The saturation model $y=y_{0}+\frac{ax}{b+x}$ was fitted to the sample data. Here, *x* denotes the antibody concentration and *y* denotes the fluorescence signal after washing. The parameter *y*_0_ was measured explicitly. The remaining parameters, i.e., the maximal fluorescence signal gain *a* and the saturation constant *b* were estimated from the data. From the maximum signal gain *a*, the corresponding concentration of bound antibody was computed from the calibration curve, i.e., $\Delta x=\frac{a}{m}$. Finally, the number of receptors per cell was computed by the formula $n=\frac{\Delta x\cdot N_{A}\cdot V}{k\cdot N_{\mathrm{cells}}\cdot M}$, where *N_A_* = 6.022 × 10^23^ 1/mol, *V*  = 50 μl, *k* = 2, *N*_cells_ = 5 × 10^5^, and *M* = 146.389 × 10^3^ g/mol denote Avogadros constant, the volume per well, the number antibody binding sites per receptor, the number of cells, and the molecular weight of the antibody. From the parameters obtained by the calibration and saturation curve, we get *n* = 122400 ± 7500. The uncertainty of the number of receptors per cell is dominated by the uncertainty of *a*, the maximum signal gain. The uncertainty of *a* is propagated to the error of *n* by Gaussian error propagation.

### 2.2 Monte-carlo simulation of observed gold cluster size distribution

The immuno-gold-staining and counting process was simulated by a Monte-Carlo approach. It is assumed that the observed gold cluster size distribution is a superposition of distributions generated by single size oligomers. Each oligomer size produces a characteristic distribution of observed gold cluster sizes that depends on the staining efficiency. The characteristic distribution ranges from exclusively monomeric observation to exclusively single size oligomeric observation for staining probabilities zero and one, respectively. The distributions in between zero and one depend on the oligomer geometry, which is reflected by the number of next neighbors of an average receptor. This number is at least 2, i.e., for linear arrangement of the receptors. For other cases, like dense circle packing resulting in a triangular geometry it is 6 and for a quadratic grid it is 8. Simulations have been performed for linear arrangements and quadratic grids which reflect different extremes. An additional factor for the observed size distribution is the gold-reagent itself, which is potentially pre-clustered. Further, the staining efficiency, i.e., the number of receptors that are stained; the geometry, i.e., the receptor positions within the oligomers being stained; and potential unspecific stainings, i.e., presence of gold particles that are not bound to any BCR, have to be considered. For given oligomer size, staining efficiency, geometry, and gold distribution, the observed gold cluster size distribution is obtained by repeated random number generation for the number of stained receptors, their positions and the number of gold particles per staining spot. For each set of random numbers, the resulting representation of the gold particle pattern is evaluated by the simulation program and the number of counted monomers, dimers, etc., is collected. This procedure was performed 10^5^ times for 10 staining probabilities between 2 and 40%, underlying BCR oligomer sizes from 1 to 40 and three geometries, i.e., linear, triangular, and quadratic. In addition, the simulation approach has been adapted to explain the observation of the gold-reagent control experiment.

### 2.3 Statistical inference

The result of each gold-staining experiment is a distribution of gold cluster sizes determined from gold particle counting in the microscope. These experimental data are compared to the simulated data and by means of statistical methods it is decided whether simulation and experiment are in accordance. We tested four major hypotheses: receptors are organized as:

1. BCR oligomers of a unique fixed size *s*,
2. BCR monomers and oligomers of a unique fixed size *s*,
3. BCR dimers and oligomers of a unique fixed size *s*,
4. BCR monomers, dimers, and oligomers of a unique fixed size *s*.

For each hypothesis, a likelihood function is derived based on the assumption of Poisson statistics for the counted gold oligomers. The corresponding log-likelihood reads

(1) $$l\left(\theta \right)=\sum_{i}m_{i}\left(\theta \right)-N_{i}\log\left(m_{i}\left(\theta \right)\right)+\overset{N_{i}}{\underset{k=1}{\sum }}\log\;k,$$

where *m_i_* is the number of expected BCR oligomers of size *i* predicted by the simulation and *N_i_* is the number of gold oligomers counted in the experiment. Minimization is performed with respect to the parameter vector θ which is defined differently for each hypothesis. This is explicitly

(2) $$\begin{array}{ll} m_{i}^{\left(1\right)}\left(\theta \right) & =\theta \cdot n_{i,sim}\left(s\right), \end{array}$$

(3) $$\begin{array}{ll} m_{i}^{\left(2\right)}\left(\theta \right) & =\theta_{1}\cdot n_{i,sim}\left(1\right)+\theta_{2}\cdot n_{i,sim}\left(s\right), \end{array}$$

(4) $$\begin{array}{ll} m_{i}^{\left(3\right)}\left(\theta \right) & =\theta_{1}\cdot n_{i,sim}\left(2\right)+\theta_{2}\cdot n_{i,sim}\left(s\right), \end{array}$$

(5) $$\begin{array}{ll} m_{i}^{\left(4\right)}\left(\theta \right) & =\theta_{1}\cdot n_{i,sim}\left(1\right)+\theta_{2}\cdot n_{i,sim}\left(2\right)+\theta_{3}\cdot n_{i,sim}\left(s\right), \end{array}$$

where *n_i,sim_*(*s*) denotes the simulated observed gold cluster size distribution given the underlying BCR oligomer size. I.e., the parameter vector θ reflects the composition of BCR oligomers on the surface. For each simulated data set, i.e., for each triplet (staining efficiency *p*, underlying cluster size *s*, geometry *g*), the maximization of the log-likelihood results in an estimate for the parameter vector θ, denoted by $\hat{\theta },$ that explains the experimental data best. In addition, to test if the log-likelihood value $l\left(\hat{\theta }\right)$ is in accordance with the data, parametric bootstrapping is employed. In parametric bootstrapping, the model prediction $m_{i}\left(\hat{\theta }\right)$ is used to generate *de novo* observation data. In our case, 10^3^ random samples have been drawn from a Poisson distribution with mean $m_{i}\left(\hat{\theta }\right)$ and have been treated like gold particle observations. For each sample, the log-likelihood is maximized again and the values are collected in a histogram approximating the asymptotic log-likelihood distribution. The original value $l\left(\hat{\theta }\right)$ is compared to different statistics of the sampled distribution, among these *p*-value and weighted distance to the mean $\Delta =\frac{l\left(\hat{\theta }\right)-\left\langle l\left(\theta \right)\right\rangle }{\sigma_{l\left(\theta \right)}}.$ The hypotheses are rejected based on these values for *p* < 0.01 and Δ > 3, respectively.

## 3 Results

### 3.1 The workflow

**Figure 1:**
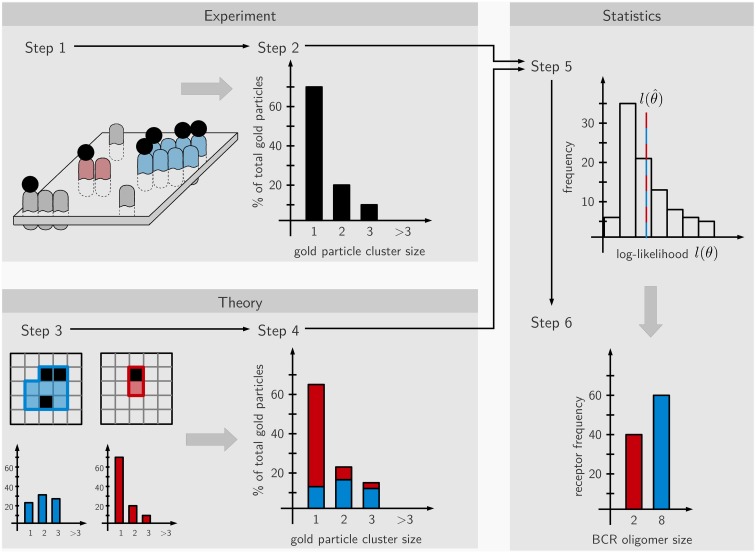
**Scheme of the workflow**. The approach is divided into six steps partitioned into experimental (steps 1 and 2), theoretical (steps 3 and 4), and statistical parts (steps 5 and 6). In step 1, the IgD-BCRs on the surface of resting and fixed B cells are stained with immuno-gold and the gold particles visualized by TEM. In step 2, the gold particles are counted, generating an observed gold cluster size distribution. In step 3, the staining process is simulated by Monte-Carlo procedures based on hypothetical BCR nanocluster size distributions, generating characteristic gold particle cluster distributions for the observed gold-labeling. Gold particles are shown in black and BCRs in blue or red (each BCR occupies one square, thus showing a blue 8 mer and a red dimer). In step 4, superposition parameters are estimated based on the observed gold particle distributions by maximum-likelihood estimation. In step 5 based on the estimated parameters, *de novo, in silico* cluster size distributions are repeatedly generated and the corresponding log-likelihood is re-optimized, providing a histogram of log-likelihood values. Step 6; if in accordance with the data, the theory makes a valid prediction for the underlying BCR nanocluster distribution and the relative amounts of the nanocluster sizes is calculated.

Here we derived the size distribution of the IgD-BCRs on the cell surface of J558L transfectants from the measured distribution of gold particles after staining the BCRs with immuno-gold. “Size distribution of the BCRs” is defined as the percentage of BCRs in a given cluster size, such as 5% of the BCRs are in BCR monomers, 45% are in BCR dimers, and 50% are in BCR trimers. Our workflow consists of two phases. The first is carried out in the wet lab and comprises immuno-gold-labeling of the BCRs on untreated and PFA-fixed cells, followed by cell surface replica preparation and the quantification of the gold particle cluster size distribution on the replicated cell surface area by TEM (Figure 1, steps 1 and 2). As the staining efficiency reached by immuno-gold-labeling is low, the obtained immuno-gold data cannot directly be converted into the distribution of the BCRs. Thus, in the second phase mathematical modeling is used to derive the BCR distribution from the gold particle data. To do so, we assume a large number of different BCR distributions, such as only one defined size (only monomers, or only dimers, or only trimers, etc.) or a combination of sizes (for example, dimers and trimers). The observed gold particle size distributions are then stochastically simulated using a Monte-Carlo simulation of the staining process, and the likelihood that the gold size distribution represents a given BCR size distribution is calculated. Each single BCR oligomer size produces a characteristic distribution of observed gold cluster sizes that depends on (1) the pre-clustering of the gold-reagent, i.e., presence of monomeric, dimeric, or trimeric gold particles in the staining reagent, (2) the immuno-gold-staining efficiency, i.e., the percentage of BCRs that are labeled by gold particles, (3) unspecific binding of the gold-reagent to the B cell, independent of any BCR, (4) the oligomer geometry which is reflected by the number of next neighbors of an average receptor within the BCR oligomer, this number is at least 2, i.e., for linear chains of receptors, or up to 8 for a quadratic grid (Figure 3A). The Monte-Carlo simulation provides information on the staining patterns and thus, the visible gold cluster size distribution (step 3). To match the data, several such gold cluster distributions are superposed with different strengths. These strengths, called the superposition parameters, are estimated from the data and give rise to a hypothesized underlying BCR size distribution (step 4). Based on the estimated superposition parameters the gold particle observation data is repeatedly generated and the parameters are re-estimated. This process results in a distribution of log-likelihood values which, in case of good agreement between model and data, contains the original log-likelihood value $l\left(\hat{\theta }\right)$ (step 5). For the log-likelihood value, the threshold corresponding to a *p*-value of 0.01 can be computed which when being exceeded allows to reject the model. Otherwise, for a model log-likelihood value in the interior of the distribution, the model makes a valid prediction for the underlying BCR oligomer size distribution (step 6). This means that the observed gold cluster distribution can be fully explained by the predicted BCR oligomer distribution.

### 3.2 Estimation of the degree of pre-clustering of the gold-reagent

**Figure 2:**
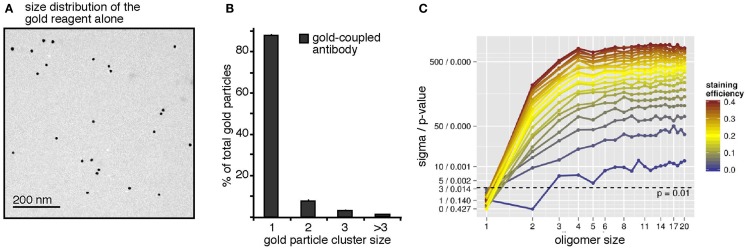
**The antibody-coupled gold-reagent is mainly monomeric**. **(A)** The gold-reagent alone was adsorbed onto collodion/carbon-coated EM grids and analyzed by TEM. **(B)** Analysis of the clustering of 2041 gold particles alone showed 88% monomeric gold particles, 8% of gold particles as close as dimers, 3% were counted as trimers, and 1% as clusters of larger sizes. **(C)** The gold-reagent cluster size distribution was re-evaluated by our workflow, interpreting the cluster counts as receptor staining. Pre-clustering of the gold-reagent was taken into account. For different assumed receptor oligomer sizes (*x*-axis) and staining efficiencies (color scale), significance level and *p*-value were computed (*y*-axis). Models are rejected above the dashed line corresponding to a *p*-value of 0.01. The analysis confirms the monomer hypothesis and rejects other hypotheses with *p* < 0.01 (above the dashed line) for staining efficiencies larger 3%.

To address a possible pre-clustering of the gold-reagent used for the BCR immuno-gold-labeling, the gold-reagent alone was adsorbed onto collodion/carbon-coated EM grids, i.e., without receptor binding, and analyzed by TEM (Figure 2A). The few observed gold particle dimers and trimers could be due to either pre-clustering of the gold particles in the staining reagent or random collocation of monomeric gold particles on the grid. The observed size distribution data of the gold-reagent alone (Figure 2B) was subjected to our workflow described above and interpreted as the staining of one huge quadratic oligomer. The size of this quadratic oligomer is the number of image pixels divided by the number of pixels per gold dot. The staining efficiency was first assessed by the number of gold particles divided by the oligomer size. Subsequently, the observed oligomer size distribution has been simulated by our Monte-Carlo approach showing that random collocation is not sufficient to explain the number of observed gold particle dimers but that the gold-reagent is indeed already pre-clustered. From this analysis, we find that 92.8% of all gold particles are monomeric, 6.5% are pre-clustered dimers, and 0.7% are pre-clustered trimers. These numbers have been taken into account for all following simulations by introducing parameters representing the inherent fraction of gold dimers and trimers. A complementary perspective on Figure 2A is its interpretation as staining of a surface with exclusively receptor monomers. This perspective enables insights into the specificity of the approach. Testing the hypothesis “100% monomers,” differences in the staining efficiency should not lead to differences in the log-likelihood because only the total number of counts but not the observed size distribution changes. In contrast, when testing alternative hypotheses such as “100% dimers” or “100% trimers,” the method should allow rejecting those hypotheses. Indeed, this was the case (Figure 2C). The plot shows the weighted distance to the mean and the *p*-value based on the log-likelihood. These values were computed for different hypothetical staining efficiencies, represented by different colors, and dominant oligomer sizes, represented by the *x*-axis. Already for hypothetical staining efficiencies larger than 3% all tested hypotheses other than “100% monomers” can be rejected with *p* ≤ 0.001. Conversely, the monomer assumption is not rejected for any staining efficiency, in accordance with the expectation. This proofs that our approach is highly sensitive as a staining efficiency of 3% is already sufficient to reject wrong hypotheses.

### 3.3 Calculation of the immuno-gold-staining efficiency for the IgD-BCR

**Figure 3:**
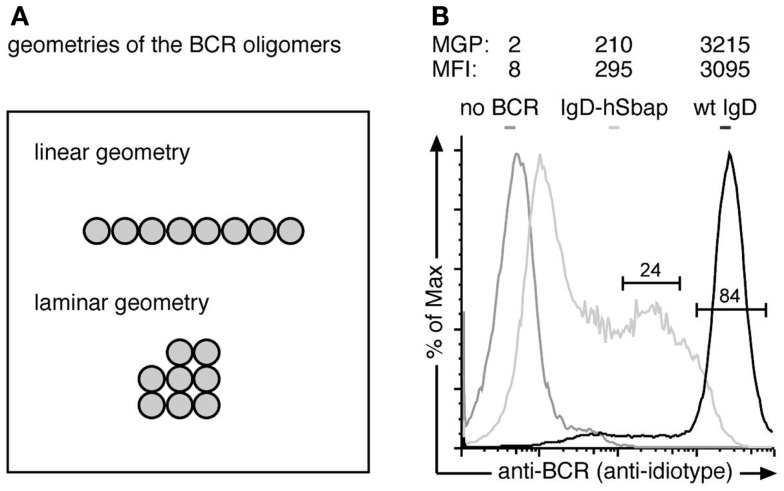
**The anti-BCR immuno-gold-labeling is specific**. **(A)** The Monte-Carlo simulations were performed for two possible BCR oligomer geometries: a linear and a laminar geometry. **(B)** J558Lδm/mb-1flN cells expressing the wt IgD-BCR (black line), J558L cells without any BCR (dark gray line) or IgD-hSbap BCR-expressing J558L (light gray line) were labeled with the anti-BCR antibody Ac146 and subsequently stained with FITC-labeled anti-mouse IgG or gold-coupled anti-mouse IgG antibodies. Fluorescently labeled cells were analyzed by flow cytometry, and mean fluorescent intensities (MFI) of the gated populations are given. Gold labeled cells were subjected to replica preparation and analyzed by TEM. The mean gold particle (MGP) number detected on the cell replicas confirms specific labeling of the BCR on wt and IgD-hSbap expressing J558L cells, while immuno-gold-labeling of J558L not expressing BCR does not result in significant gold particle detection on surface replicas.

Next, we calculated the staining efficiency of the BCR immuno-gold-labeling process, using the murine B cell line J558Lm/mb-1flN (16). These cells express IgD-BCRs on their surface, as seen by a flow cytometric analysis using the same monoclonal anti-BCR antibody (Ac146) that was also used for the immuno-gold-labeling (Figure 3B). The total number of IgD-BCRs per cell was experimentally determined to be 122400 ± 7500 BCRs (described in the Methods section). The total cell surface area of a J558L cell is 782 ± 25 μm^2^ (25) resulting in an expected mean density of 156 BCRs/μm^2^. We analyzed three J558Lδm/mb-1flN cells by immuno-gold-staining and TEM. The three areas analyzed by TEM were 101, 183, and 152 μm^2^ and the expected BCR number in those areas was calculated to be 15600, 28080, and 23400 BCRs, respectively. The achieved BCR immuno-gold-labeling efficiency was calculated based on the number of gold particles observed in the areas (2192, 3577, and 3876 gold particles, respectively) assuming one gold particle to represent one BCR. Thus, the BCR labeling efficiencies were 14.1, 12.7, and 16.6%, respectively. Thus, the BCR staining efficiency in our experiment was approximately 15%.

### 3.4 The anti-BCR immuno-gold-staining is specific for the BCR

To prove that the anti-BCR immuno-gold-labeling protocol only stained BCRs, we compared J558L cells that lack BCR expression and the J558Lδm/mb-1flN cells (Figure 3A). Flow cytometry analysis confirmed no BCR expression on J558L cells. Immuno-gold-labeling of the J558L cells resulted in 2 gold particles per observed area, while J558Lδm/mb-1flN cells were stained with 3215 gold particles on average (Figure 3A). Thus (nearly) each gold particle is representing a BCR.

### 3.5 Determination of the size distribution of wt IgD-BCR oligomers

**Figure 4:**
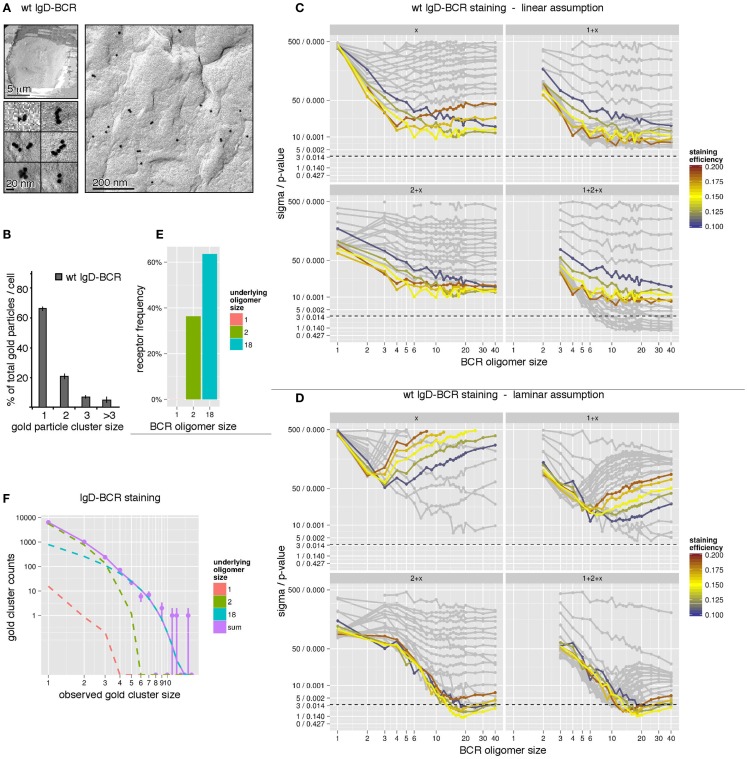
**The IgD-BCR forms differently sized oligomers on the surface of B cells**. **(A)** IgD-BCR-expressing J558Lm/mb-1flN cells were immuno-gold stained using the mouse anti-BCR antibody Ac146 and anti-mouse IgG antibodies coupled to gold particles of 10 nm. The cell overview is given at low magnification (upper left panel), gold-labeling of the cell surface is shown in an overview picture (right panel) and individual gold clusters are shown at high magnification (lower left panel). **(B)** 66% of the gold particles were present as monomers, 21% formed dimers, 7.5% trimers, and 5.5% were present in clusters of sizes larger than 3. **(C)** The observed gold particle cluster size distribution was simulated with the assumption of an underlying linear BCR oligomer geometry. Simulations were performed for staining efficiencies of 15 ± 5% indicated by colored lines. The BCR oligomer sizes tested are indicated on the *x*-axis, and the sigma/*p*-values for the statistical analysis of each simulation set is given on the *y*-axis. Different hypothetical BCR oligomer size distributions are tested, namely “one BCR oligomer size only” (*x*, upper left panel), “BCR monomers + another single BCR oligomer size” (1 + *x*, upper right panel), “BCR dimers + another single BCR oligomer size” (2 + *x*, lower left panel), and “BCR monomers, dimers + another single BCR oligomer size” (1 + 2 + *x*, lower right panel). Models are rejected above the dashed line corresponding to a *p*-value of 0.01. **(D)** The observed gold particle cluster size distribution was simulated as in (C) with the assumption of an underlying laminar BCR oligomer geometry. **(E)** Our simulations predict that 60% of all receptors are part of oligomers of size 18 in a laminar geometry and 40% are BCR dimers. **(F)** Gold cluster counts (dots with error bars) compared to the prediction of best fitting model (continuous line). The number of counts originating from the different underlying BCR oligomer sizes are shown as dashed lines, indicating that observed clusters up to a size of 3 are primarily caused by BCR dimers, while gold clusters larger than 3 can be explained by BCR clusters of size 18. The impact of BCR monomers is negligible.

For our simulations to determine the size distribution of the wt IgD-BCR, we have used the immuno-gold data from J558Lδm/mb-1flN cells (Figure 4A) and the corresponding size distribution of the gold particles (Figure 4B). The quantification of gold clusters by TEM revealed 66% gold monomers, 21% gold in dimers, 7.5% gold in trimers, and a fraction of 5.5% gold particles were part of oligomers of four or more gold particles. To account for different possible geometries of each individual BCR cluster, simulations have been performed for linear and laminar BCR arrangements (Figure 3B). Firstly, we have assumed a linear arrangement of the BCRs (Figure 4C). Assuming the presence of one defined BCR oligomer size, labeled by *x*, we have performed simulations for BCR monomers (1), BCR dimers (2), BCR trimers (3), etc. (Figure 4C, top, left panel). The different staining probabilities are denoted by the color coding being centered around the experimentally measured value of 15%. All curves outside a range of ±5% were shaded. For the single size assumption none of the assumed BCR cluster sizes is in agreement with the data (below the dotted line). The discrepancy between data and model does not change any more as soon as the assumed BCR oligomer size is larger than 6 because the observed size distribution produced by them is almost identical. In particular, this means that linear BCR oligomers larger than 6 or even a mixture of large BCR oligomers is not in accordance with the experimental data at the given staining efficiency. Also a mixture of small and large BCR oligomer sizes, e.g., 1 + *x* (BCR monomers and one BCR cluster size of *x*, upper, right panel), 2 + *x* (BCR dimers and one BCR cluster size of *x*, lower, left panel), or 1 + 2 + *x* (BCR monomers, dimers, and one BCR cluster size of *x*, lower, right panel), is unable to explain the observed data by linearly arranged receptor oligomers, thus, leading to a full rejection of this geometry hypothesis. Secondly, we have assumed a laminar arrangement of the BCRs (Figure 4D). Following the plot for the single size assumption (Figure 4D, panel *x*), the best agreement is obtained for small BCR oligomers such as BCR dimers and trimers. This suggests that small BCR oligomers contribute significantly to the overall observed gold cluster distribution. Evaluation of related model hypotheses, 1 + *x*, 2 + *x*, and 1 + 2 + *x* shows that monomers plus an additional BCR oligomer size is not sufficient to explain the observed size distribution. However, accordance can be achieved for BCR dimers plus a large BCR oligomer around a size of 18 (lower, left panel). Additional BCR monomers do not further reduce the discrepancy between model and data (lower, right panel). This is also expressed in Figure 4F. BCR dimers dominate the small observed gold particle oligomers up to a size of 3, where BCR dimers are occasionally observed as trimers due to the pre-clustering of the gold-reagent. Underlying BCR oligomers of size 18 take over beginning from an observed gold particle size of 4. The contribution by BCR monomers is negligible. When testing a model with several BCR oligomer sizes, e.g., 1 + 2 + 18, the maximization of the log-likelihood gives an estimate for the ratio between the abundance of the single BCR oligomer sizes. This ratio is then expressed in the percentage of receptors being contained in either of the oligomers. The number of receptors in oligomers of each size, computed from the model, indicates that 60% of all receptors are contained in BCR oligomers of size 18, 40% are contained in BCR dimers and BCR monomers are negligible (Figures 4E,F). In conclusion, we suggest that IgD-BCRs are arranged in pre-formed BCR oligomers on the surface of J558Lδm/mb-1flN B cells and that BCR dimers as well as large BCR oligomers of a laminar geometry co-exist.

### 3.6 Determination of the size distribution of a mutant IgD-BCR that mostly forms dimers

**Figure 5:**
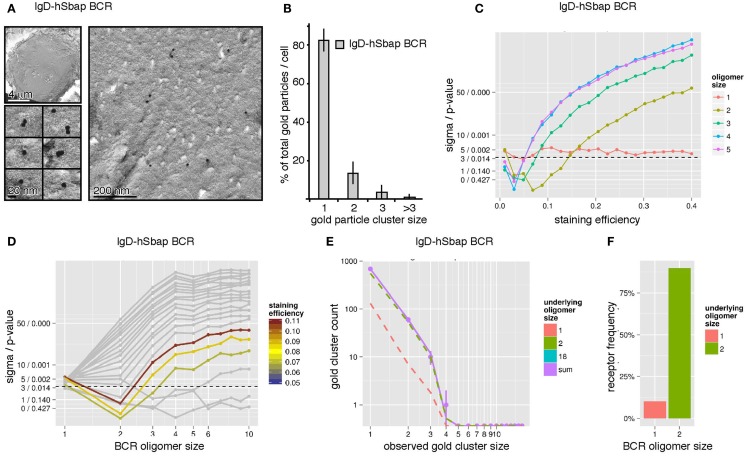
**The IgD-hSbap BCR mutant forms mainly BCR dimers on the surface of B cells**. **(A)** IgD-hSbap BCR mutant-expressing J558L cells were immuno-gold stained and analyzed as in Figure 4A. **(B)** 83% of the gold particles were monomeric, 13% formed dimers, and 4% were present in clusters of sizes larger than 2. **(C)** The observed gold particle cluster size distribution was simulated for different BCR staining efficiencies (*x*-axis) and different underlying BCR oligomer sizes (color scale). The sigma/*p*-values for the statistical analysis of each simulation set are given on the *y*-axis. BCR dimers with a staining efficiency of 8% are identified as the most likely explanation for the observed gold cluster distribution. **(D)** Simulations were performed as in **(C)**. Assuming a staining efficiency of approximately 8% (color coding), sigma/*p*-values (*y*-axis) are plotted for the different BCR oligomer sizes (*x*-axis). **(E)** Gold cluster counts (dots with error bars) compared to prediction of best fitting model (continuous line). The number of counts originating from monomers and dimers are shown as dashed lines, indicating that the observed distribution is dominated by BCR dimers. **(F)** A model with BCR monomers and dimers was fitted to the data, predicting that 90% of all receptors are part of dimers, 10% are monomers.

To validate our combined experimental and theoretical approach, we took advantage of a transmembrane mutant IgD-BCR (IgD-hSbap BCR), which resulted in impeded BCR oligomerization as detected by Blue Native gel electrophoresis and pre-dominant detection of BCR dimers (16). Immuno-gold-labeling of the mutant BCR and sampling were performed as above. The quantification of gold clusters by TEM revealed mainly gold monomers (83%) and dimers (13%), and a smaller fraction of gold oligomers of three or more gold particles (4%) (Figures 5A,B). In contrast to the wt IgD-BCR, the staining efficiency was not determined as mutant IgD-BCR expression was heterogeneous (Figure 3B). When evaluating the log-likelihood for BCR staining efficiencies up to 40% and dominant BCR oligomer sizes up to10, the overall best log-likelihood value was achieved for BCR dimers at a staining efficiency of 8.8% (Figure 5C). The efficiency value is not too far from the wt IgD-BCR staining efficiency, which has been measured to be 15%. Our Monte-Carlo simulation indicated that IgD-hSbap BCR oligomers larger than two can be rejected at high confidence level unless the staining efficiency would be lower than 5% (Figure 5D). Also BCR monomers without larger BCR oligomers cannot explain the data, thus, confirming that the mutant BCR dimerizes. In addition to the single size model, a model with monomers and one further BCR oligomer size was tested. The observed gold cluster distribution after immuno-gold-labeling of the mutant IgD-BCR together with the model prediction of the best fitting model, i.e., monomers plus dimers, is shown in Figure 5E. Analogously to the wt IgD-BCR experiment, the percentage of IgD-hSbap BCR present as BCR monomers and BCR dimers was computed, demonstrating that 90% of the mutant BCRs are present as dimers and 10% as monomers (Figure 5F). These findings validate our approach and reflect the Blue Native gel electrophoresis data for IgD-hSbap BCR (16).

## 4 Conclusion

Here, we used a combined immuno-gold TEM and modeling approach to suggest that the IgD-BCR is expressed as BCR dimers and larger oligomers. To measure the distribution of cell surface molecules super resolution light microscopy techniques, such as high-speed photoactivated localization microscopy (5) or dSTORM (21), have been employed. Unlike these light microscopy techniques, our technique permits nano-scale resolution of cell surface molecules on the membranes that are not in contact with any support, thereby avoiding visualization of potential clustering artifacts induced by cell adherence (26). This advantage is not given in other EM methods, such as nano-probe labeling and transmission microscopy of cytoplasmic face-up sheets of cell membrane ripped off from cells to generate cytoplasmic face-up membrane sheets (27), which subsequently can be fixed and immuno-gold labeled (5, 28, 29). Our method can measure the distribution of any cell surface molecule, without the need to genetically modify this protein with a fluorophore, provided that antibodies against the protein are available. It is, however, not compatible with dynamic observations and, given the relatively low labeling efficiency, does not allow absolute quantifications by direct gold counting; here we present an approach to overcome the latter limitation. We assumed that the presence of one gold particle is indicative of one BCR (with the restraint that some gold particles are aggregated, which we have taken into account). There is the unlikely possibility that our staining approach with a primary anti-BCR and a secondary gold-coupled antibody could allow for double labelings of the BCRs, if the secondary antibody binds twice to the primary antibody (once to each of the two heavy chains). However, double labeling most likely did not take place: we used the IgD-hSbap BCR that only formed monomers and dimers when analyzed by Blue Native gels (16). And indeed, in our analysis this mutant BCR was exclusively detected as monomers (10% of the BCRs) and dimers (90% of the BCRs). These relative abundancies fit well to the biochemical Blue Native gel data (16). Thus, we conclude that a potential double gold-labeling of a BCR does not take place in our experiments and does not prevent drawing our conclusions from the immuno-gold TEM data. Here, we estimate that 60% of all wild type IgD-BCRs expressed on J558Lδm/mb-1flN cells are part of BCR oligomers of a size of around 18 and 40% form BCR dimers. This notion is based on the most simple BCR size distribution (i.e., assuming the lowest number of different BCR sizes) to explain the observed gold distribution. Still, the co-existence of larger number of different BCR oligomer sizes cannot be ruled out. Previously, the oligomer size of the IgM-BCR has been theoretically approached by Iber and Gruhn (30). Assuming oligomer formation and decay rates based on the literature, they calculated that IgM-BCR pentamers might exist. However, the modeling approach was not based on experimental data on BCR oligomer sizes and thus the presence of BCR pentamers is rather hypothetical. BCR pre-clustering might increase the antigen-receptor avidity, since functional antigens are mostly multimeric structures (31, 32). This increase in avidity could enhance the sensitivity of B cell activation, as suggested for TCR pre-clusters (8–10). BCR oligomers could also enhance the sensitivity of B cells by cooperativity between BCRs in one oligomer. Thus, antigen-binding to one or two BCRs could also lead to the activation of non-engaged BCRs, propagating the signal within one oligomer. In line with this, we had suggested earlier that the kinase Syk bound to one BCR can phosphorylate the neighboring BCR amplifying BCR signaling by a positive feedback loop (33). In T cells it was shown experimentally that individual TCRs cooperate in this manner within one TCR pre-cluster (34). On the other hand, enhanced sensitivity of the B cells might also bear an increased risk of reacting against abundant self-antigens and lead to autoimmunity. The presence of pre-formed BCR oligomers has important implications for the mechanism of how the BCR transmits the signal of antigen-binding into the interior of the cells. Initially, it was assumed that the BCRs are individually distributed on the B cell surface and only brought into close proximity by their multivalent antigens, leading to reciprocal phosphorylation of the cytoplasmic tyrosines in Igα and Igβ and thereby signal initiation. The idea of cross-linking as the first step in BCR activation was based on the finding that monovalent antigens do not induce BCR activation (31, 32). Now, the cross-linking model is questioned by the finding that BCR oligomers exist (16, 17, 19, 21, 35). In our analysis we hardly detected any BCR monomers, which is in line with the finding that only oligomerized are stably expressed on the cell surface (19). Our study clearly points out that BCR oligomers of different sizes co-exist on the surface of resting B cells. Activation therefore might occur according to conformational changes within the BCR oligomer, as e.g., the proposed in the dissociation activation model (20). Knowledge on the pre-clustering of a given receptor not only aids to explain the biology of the receptor, but also might open new strategies for interfering with its function for vaccination or therapeutic purposes.

## Conflict of Interest Statement

The authors declare that the research was conducted in the absence of any commercial or financial relationships that could be construed as a potential conflict of interest.
